# Associations between Longer Habitual Day Napping and Non-Alcoholic Fatty Liver Disease in an Elderly Chinese Population

**DOI:** 10.1371/journal.pone.0105583

**Published:** 2014-08-20

**Authors:** Hua Qu, Hang Wang, Min Deng, Huili Wei, Huacong Deng

**Affiliations:** Department of Endocrinology, the First Affiliated Hospital of Chongqing Medical University, Chongqing, China; National Institutes of Health, United States of America

## Abstract

**Context:**

Both longer habitual day napping and Non-Alcoholic Fatty Liver Disease (NAFLD) are associated with diabetes and inflammation, but the association between day napping and NAFLD remains unexplored.

**Objective:**

To investigate the association between the duration of habitual day napping and NAFLD in an elderly Chinese population and to gain insight into the role of inflammatory cytokines in this association.

**Design and Setting:**

We conducted a series of cross-sectional studies of the community population in Chongqing, China, from 2011 to 2012.

**Participants:**

Among 6998 participants aged 40 to 75 years, 6438 eligible participants were included in the first study and analyzed to observe the association between day napping duration and NAFLD. In a separate study, 80 non-nappers and 90 nappers were selected to identify the role of inflammatory cytokines in this association. Logistic regression models were used to examine the odds ratios (ORs) of day nap duration with NAFLD.

**Results:**

Day nappers had a significantly higher prevalence of NAFLD (*P*<0.001). Longer day napping duration was associated in a dose-dependent manner with NAFLD (*P* trend <0.001). After adjustment for potential confounders, the ORs were 1.67 (95% CI 1.13–2.46) for those reporting 0.5–1 h and 1.49 (95% CI 1.01–2.19) for those reporting >1 h of day napping compared with individuals who did not take day naps (all *P*<0.05). Longer-duration day nappers had higher levels of IL-6 and progranulin (PGRN) but lower levels of Secreted frizzled-related protein-5 (SFRP5, all *P* trend <0.001). After adjusting for IL-6, PGRN, and SFRP5, the association between day napping duration and NAFLD disappeared (all *P*>0.05).

**Conclusion:**

Longer day napping duration is associated with a higher prevalence of NAFLD, and inflammatory cytokines may be an essential link between day napping and NAFLD.

## Introduction

Non-alcoholic Fatty Liver Disease (NAFLD) is considered the most common chronic liver disease worldwide. NAFLD encompasses a disease spectrum ranging from simple steatosis to non-alcoholic steatohepatitis (NASH), and may progress to fibrosis, cirrhosis and even hepatocellular carcinoma and liver failure [1], [2]. Estimated rates of its current prevalence range from 24% to 42% in Western countries and 5% to 40% in Asian countries [3], [4]. Day napping or afternoon napping (siesta) is a common custom in many countries, particularly in the Mediterranean and Latin American countries, and the prevalence of day napping increases with age [5]. It is also a prevalent habit in China, from children to the elderly, and is considered a healthy habit in traditional conventions. Fang et al. [6] indicated that the prevalence of habitual day napping among Chinese individuals aged 45 or older was 68.6%.

Recently, a number of cross-sectional studies and prospective studies with follow-up periods of 2–10 years have concluded that daytime napping was associated with a higher risk of diabetes [7]–[9]. Evidence from the Guangzhou Burbank [10] and the Dongfeng–Tongji cohort of retired workers [6] also demonstrated that the duration of day napping was positively associated with an increased risk for type 2 diabetes among residents aged 45 years or older in China. Additionally, epidemiological studies have found a high prevalence of NAFLD in diabetic patients ranging from 50% to 75% [11]–[13], and chronic inflammation and insulin resistance were believed to be their common mechanism. However, there are no reports on the direct relationship between day napping and NAFLD to our knowledge, despite evidence showing the strong association between day napping and diabetes, and diabetes is thought to have a strong association with NAFLD.

Previous research has demonstrated that the pro-inflammatory cytokines, such as, IL-6, TNF-α, and leptin increased and that anti-inflammatory factors such as adiponectin decreased in excessive daytime sleepers [14], additionally, NAFLD is considered a chronic inflammatory disease. Therefore, we hypothesize that inflammatory cytokines may be an essential link between day napping and NAFLD.

Thus, the aim of our study is to reveal the association between day napping and NAFLD in an elderly Chinese population, and to assess whether this association is affected by inflammatory factors.

## Methods

### Study design and population

We performed two cross-sectional studies between June 2011 and December 2012 in Chongqing, China. In the first study (cohort 1), 5 districts were randomly selected from Chongqing municipality, which comprises 40 districts and counties, and 10 communities were randomly selected (2 from each district) from these 5 districts. All elderly people (aged 40 to 75 years) who were registered as residents of the selected communities were informed of the study and invited to participate. This part of the study is part of the risk evaluation of cancers in Chinese diabetic individuals: a longitudinal study (REACTION study). The rationale, design, and methods of the REACTION study have previously been described in detail [15]. In the second study (cohort 2), age-matched subsets of 80 non-nappers and 90 habitual day nappers were recruited from another community (except for the 10 communities that were selected in cohort 1) in Chongqing, China.

A total of 7168 individuals participated in these two cohorts, 6998 of these participants were included in cohort 1. Of the 6998 subjects, 560 were excluded for the following reasons: previous or present diagnoses of hepatitis B or C infection, biliary diseases, surgical interventions and other chronic liver diseases (n = 207); missing values on hours of day napping and nocturnal sleeping (n = 55); and fewer than 5 hours of nocturnal sleep duration, to avoid compensatory day napping (n = 298). After excluding these subjects, cohort 1 had a total of 6438 eligible subjects.

In each community, trained staff collected data according to a standard protocol at local community clinics. A standard questionnaire that collected information on demographics, medications, self-reported medical history, and lifestyle was administered face to face by trained investigators. Blood samples, which were used to detect plasma glucose and other parameters, were taken from all participants who had fasted for at least 10 h. Written informed consent was obtained from all participants. The study was approved by the Ethics Committee of Shanghai Jiaotong University, Shanghai, China, and the first affiliated hospital of Chongqing medical university, Chongqing, China.

### Ascertainment of diabetes and NAFLD

75 g oral glucose tolerance test (OGTT) was performed in all subjects. The diagnoses of type 2 diabetes were based on the 1999 diagnostic criteria of the World Health Organization (WHO).

NAFLD was diagnosed based on the following criteria [16], [17]:

1. Fatty liver index (FLI) >60 (sensitivity is 61%, specificity is 86%). The FLI was first established by Bedogni et al. in 2006 and had been tested for its sensitivity and specificity by ultrasound in different populations [18], [19].
2. Absence of all other causes of chronic liver disease, e.g., previous or present diagnoses of hepatitis B or C infection, biliary diseases, surgical interventions and other chronic liver diseases (autoimmune, celiac disease, genetic disorders such as Wilson’s disease and α-1-antitrypsin deficiency) based on self-reports.
3. No history of current or past excessive alcohol consumption, defined as average daily consumption of alcohol >20 g/day (140 g/week) in males and > 10 g/day (70 g/week) in females based on self-reported frequency and daily amount of alcohol consumption.
4. No history of systemic illness known to cause fatty liver disease;

### Assessment of habitual day napping and nocturnal sleep

Habitual day napping was defined as taking a planned or regular nap as a habit more than three times per week after lunch over the past 12 months. Individuals who used compensatory daytime sleeping because of nocturnal sleep deprivation for any reason were excluded as habitual day nappers. Habitual day napping was assessed by asking the subjects “Do you have a habit of taking a nap after lunch?” Those who answered yes were further asked about the frequency, quality, and duration of their naps. Subjects were also asked to self-report their usual lengths of nocturnal sleep time and when they woke up in the morning; the duration of nocturnal sleep was thereby calculated.

### Assessment of demographic and lifestyle information

The standard questionnaire collected demographic information on sex, age, education (e.g., primary school or below, junior high school, high school, college or above), and previous or current physician-diagnosed diseases including liver disease, hypertension, coronary heart disease, myocardial infarction, stroke and tumor. We also asked questions (kind and frequency) about the drugs that the subjects had taken over the previous six months. Using the questionnaire, we also collected the participants’ lifestyle information, such as past or current cigarette smoking, past or current alcohol drinking, snoring, and walking time per week (h/w).

### Clinical evaluation

Standardized protocols were used to measure the height, body weight, waist circumference, hip circumference, and blood pressure (BP) in all subjects. Height, waist and hip circumference were measured to the minimum recorded unit of 0.1 cm; body weight was measured to an accuracy of ±0.1 kg; and blood pressure was measured using an automated electronic device (OMRON Model HEM-725 FUZZY, Omron Company, Dalian, China) on the nondominant arm of seated participants three times consecutively at 1-minute intervals after a ≥5-minute rest. The three readings were averaged for the analysis. Body mass index (BMI) and waist-to-hip ratio (WHR) were calculated.

Overnight fasting blood samples were collected to determine the fasting plasma glucose (FPG), HbA1c, fasting insulin (FINS), triglyceride (TG), total cholesterol (TC), high-density lipoprotein cholesterol (HDL-c), low-density lipoprotein cholesterol (LDL-c), liver and kidney function. Blood samples were also collected after 2 hours of a 75 g OGTT to determine the 2 h plasma glucose (2 hPG). All of the blood samples were separated within 1 h, and then frozen at −80° until they were used in this study, all within 3 months. Glucose was assayed using the glucose oxidase method. HbA1c was determined using the method of high-performance liquid chromatography (VARIANT™ II and D-10™ Systems, BIO-RAD, USA). Fasting insulin (FINS) was measured using an autoanalyzer (ARCHITECT i2000SR System, Abbott Laboratories, IL, USA). Lipid profiles and liver and kidney functions were detected using a biochemical autoanalyzer (ARCHITECT c16000 System, Abbott Laboratories, IL, USA). Insulin resistance was evaluated using the homeostatic model (HOMA-IR).

### Assessment of plasma progranulin, IL-6, and SFRP5 concentrations in cohort 2

Plasma progranulin (PGRN), IL-6, and Secreted frizzled-related protein-5 (SFRP5) concentrations were determined by enzyme-linked immunosorbent assays according to the manufacturers’ instructions (Human ELISA kit, CUSABIO Science Co, Ltd, China). All samples were run in duplicate and repeated if there was a >15% difference between duplicates. No significant cross-reactivity or interference was observed.

### Related calculation formulas

Body mass index (BMI) formula is weight in kilograms divided by height in meters squared.

The homeostasis model assessment for insulin resistance (HOMA-IR) as computed as follows: Fasting insulin (mU/L)×Fasting plasma glucose (mmol/L)/22.5.

Fatty liver index (FLI) was calculated as follows: (e^0.953*ln (triglycerides, mg/dL)+0.139*BMI (kg/m2)+0.718*ln (ggt, U/L)+0.053*waist circumference (cm)−15.745^)/(1+ e^0.953*ln (triglycerides, mg/dL)+0.139*BMI (kg/m2)+0.718*ln (ggt, U/L)+0.053*waist circumference (cm) −15.745^)*100.

### Statistical analysis

SPSS software, version 19.0 (IBM, Armonk, NY), was used for all statistical analyses. Data are expressed as median (interquartile range, IQR, 25%–75%) for non-normally distributed continuous variables and proportions for categorical variables. Differences between groups were tested using ANOVA for continuous variables, and Chi-square test was used to test for differences in the distribution of categorical variables. Nonparametric methods were carried out for non-normally distributed values. Multivariate odd ratios (ORs) and 95% confidence intervals (CIs) were derived from logistic regression models, in cohort 1, we used NAFLD as dependent variables to analyse the association between afternoon nap duration with NAFLD. participants Reporting no napping were used as the reference group and those with 1 h and more of napping were grouped together because only 1% of the participants reported >2 h of napping. Covariates included age, sex, education, snoring, past and current smoking status, past and current alcohol drinking status, BP, Nighttime sleep duration, walk time, WHR, BMI, HOMA-IR, diabetes. We fitted the regression models with these variables individually or simultaneously. The linear trend was estimated by linear-by-linear association of chi-square test. In cohort 2, each inflammatory factor was divided into tertiles for the linear trend was test. In all statistical tests, P values<0.05 were considered as significant.

## Results

### Characteristics of participants in cohort 1 and 2

**Figure 1:**
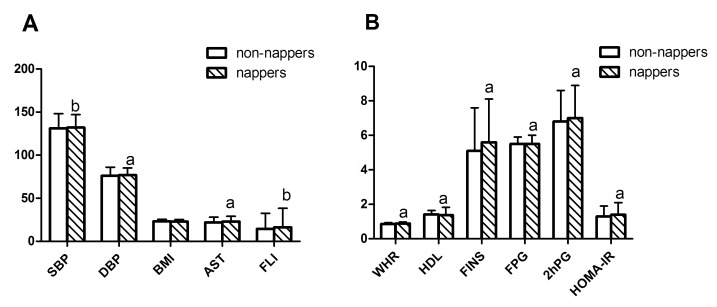
Parameters in cohort 1 population according to whether they take day naps. Data are present as median (IQR). ^a^ *P*<0.01 compared with non-nappers, ^b^ *P*<0.05 compared with non-nappers.

In cohort 1, 6438 participants were recruited (median age, 61 years; IQR, 51–67 years), of which, 1489 participants (23.1%) reported regularly taking day naps. Table 1 shows the population characteristics according to whether they took day naps. Compared with the participants who reported no napping, nappers were more likely to report snoring (36.7% versus 43.5%, *P*<0.001), 7–8 h of night sleep (39.5% versus 43.3%, *P* = 0.010), and higher levels of education (*P* for trend <0.001). After examination, more nappers were diagnosed as DM (1.17 fold higher prevalence compared with non-nappers), and they were more likely to have higher levels of SBP, DBP, TG, AST, fasting insulin, WHR, FPG, 2 hPG, HOMA-IR, and FLI (Figure 1, all *P*<0.05). The nappers had lower HDL levels (Figure 1B, *P*<0.001), and they reported less walking time than did non-nappers (*P* = 0.048). There were no differences in terms of sex, past or current smoking, and past or current drinking between nappers and non-nappers (all *P*>0.05). The levels of BMI, Cr, LDL, TC, ALT, GGT, and HbA1c were also not significantly different between nappers and non-nappers (all *P*>0.05).

**Table 1:** Population characteristics according to habitual day napping.

	Non-napper	Napper	*P*		Non-napper	Napper	*P*
n†	4949	1489		Diabetes (%)	15.1	17.7	0.015*
Age(years)	60.0	61.0	0.107	SBP(mmHg)	131.0	132.0	0.033*
	(50–60)	(53–67)			(118.0–148.0)	(117.0–147.0)	
Sex			0.972	DBP(mmHg)	76.0	77.0	0.002*
					(69.0–86.0)	(68.0–85.0)	
Men(%)	32.2	32.2		BMI(kg/m^2^)	23.3	23.1	0.464
					(21.1–25.6)	(20.7–25.4)	
Women(%)	67.8	67.8		Cr(µmol/L)	64.4	64.8	0.367
					(58.3–72.7)	(58.7–73.4)	
Education			<0.001*	HDL(mmol/L)	1.41	1.37	<0.001*
					(1.19–1.64)	(1.14–1.82)	
Elementary or below(%)	55.9	49.6		LDL(mmol/L)	2.6	2.6	0.975
					(2.1–3.2)	(2.2–3.2)	
Junior high school(%)	28.7	30.6		TC(mmol/L)	4.7	4.7	0.812
					(4.1–5.4)	(4.1–5.4)	
High school(%)	11.0	14.5		TG(mmol/L)	1.2	1.2	0.043*
					(0.8–1.6)	(0.9–1.8)	
College or above(%)	4.4	5.2		GPT(U/L)	15.0	15.0	0.831
					(11.0–20.0)	(11.0–21.0)	
Current smokers(%)	18.3	18.4	0.536	AST(U/L)	22.0	23.0	0.002*
					(18.0–28.0)	(19.0–29.0)	
Past smokers(%)	20.5	18.8	0.484	GGT(U/L)	18.0	18.0	0.207
					(13.0–28.0)	(13.0–29.0)	
Current alcohol drinkers(%)	24.2	25.1	0.639	HbA1C(%)	5.8	5.8	0.508
					(5.5–6.0)	(5.5–6.1)	
Past alcohol drinkers(%)	29.4	29.6	0.649	HbA1C(mmol/mol)	40	40	0.508
					(37–42)	(37–43)	
Snoring(%)	36.7	43.5	<0.001*	FINS(mU/L)	5.1	5.6	<0.001*
					(3.5–7.6)	(3.7–8.1)	
Night sleeping(%)			0.022*	WHR	0.86	0.89	<0.001*
					(0.81–0.93)	(0.86–0.97)	
5–6 h	22.3	22.6	0.607	FPG(mmol/L)	5.5	5.5	0.003*
					(5.1–5.9)	(5.1–6.0)	
7–8 h	39.5	43.3	0.010*	2 hPG(mmol/L)	6.8	7.0	0.002*
					(5.7–8.6)	(5.9–8.9)	
>9 h	38.2	34.0	0.009	HOMA-IR	1.3	1.4	<0.001*
					(0.8–1.9)	(0.9–2.1)	
Walk time(h/w)	6.0	3.0	0.048*	FLI	14.5	16.4	0.034*
	(4.8–10.5)	(2.0–7.0)			(6.3–32.5)	(6.5–38.4)	

Data are expressed as median (interquartile range, 25%–75%) for continuous variables and proportions for categorical variables. BMI, body mass index; WHR, waist hip ratio; SBP, systolic blood pressure; DBP, diastolic blood pressure; Cr, creatinine; GPT, glutamic-pyruvic transaminase; AST, aspartate aminotransferase; GGT, gamma-glutamyl transpeptidase; FPG, fasting plasma glucose; 2 hPG, 2 h postchallenge plasma glucose; FINS, fasting serum insulin; HOMA-IR, Homeostasis Model Assessment for insulin resistance; TC, total cholesterol; TG, triglyceride; HDL-c, high-density lipoprotein-cholesterol; LDL-c, low-density lipoprotein-cholesterol.

*P<0.05 compared with non-nappers.

† N = 6438.

In cohort 2, 80 non-nappers and 90 nappers were included (66 males and 104 females, mean age 60.34±6.98, range from 41–75) and were categorized into four groups (non-nappers, <0.5 h, 0.5–1 h, >1 h). There were no differences in terms of age, FPG, 2 hPG, HbA1c, HDL, LDL, TG, TC among these four groups (all *P*>0.05, data are not shown). While the FLI and BMI levels were higher in nappers with longer day napping duration (>1 h) compared with non-nappers (*P*<0.001).

### Prevalence of NAFLD by day napping duration in both cohorts

Based on day napping duration, the participants were categorized into four groups (Table 2, non-nappers, <0.5 h, 0.5–1 h, >1 h). In cohort 1, the proportion of NAFLD in longer day napping duration (>1 h) was 1.72 fold higher compared with shorter naps (<0.5 h) and 1.92 fold higher than those in non-naps (*P* for trend <0.01). In cohort 2, the proportion of day napping was 52.9%. Although the prevalence of day napping was more than twice as cohort 1, we still found a higher prevalence of NAFLD in longer day napping duration than those in shorter naps (*P* for trend <0.01).

**Table 2:** Prevalence of NAFLD according to the day napping duration.

	duration of day napping (h)				*χ^2^*	*P*_trend_
	No napping	<0.5	0.5–1	>1		
Cohort 1	431(8.7)	45(9.7)	64(11.1)	75(16.7)	38.15	<0.001
Cohort 2*	9(11.3)	5(29.4)	14(33.3)	15(48.4)	29.39	<0.001

Data are n(%). N of cohort 1 is 6438 and for cohort 2 is 170.

*cohort 2 including 80 non-nappers and 90 nappers.

### Associations between day napping duration and NAFLD

To test the association between day napping duration and NAFLD, we set NAFLD as the dependent variable. We first added basic model covariates, i.e., sex, age, education, snoring, past or current smoking, past or current drinking, SBP, DBP, and night sleep duration, and found that a longer day napping duration (0.5–1 h, OR, 1.51 [95% CI, 1.05–2.18] and >1 h, OR, 1.96 [95% CI, 1.36–2.82]) was related to a higher prevalence of NAFLD compared with the non-nappers. However, there was no significant higher risk for the shorter nappers (<0.5 h, OR, 1.17 [95% CI, 0.76–1.78]). Then, we gradually add the covariates of walking time, HOMA-IR, WHR, BMI, and diabetes. The ORs for the longer day napping duration (0.5–1 h, OR, 1.67 [95% CI, 1.13–2.46], and >1 h, OR, 1.49 [95% CI, 1.01–2.19]) were moderately attenuated after adjusting for these covariates, but they still had statistical significance (Table 3).

**Table 3:** ORs (95% CI) of NAFLD according to duration of day napping.

	Duration of napping (h)			
	None	<0.5	0.5–1	>1
Basic model*	1.00	1.17(0.76–1.78)	1.51(1.03–2.20)	1.96(1.36–2.82)
+walk time	1.00	1.17(0.76–1.78)	1.51(1.05–2.19)	1.96(1.36–2.82)
+walk time, HOMA-IR	1.00	1.22(0.79–1.87)	1.54(1.06–2.24)	2.00(1.38–2.90)
+walk time, HOMA-IR, WHR	1.00	1.18(0.77–1.81)	1.46(1.01–2.14)	1.92(1.32–2.79)
+walk time, HOMA-IR, WHR, BMI	1.00	1.18(0.77–1.81)	1.46(1.01–2.14)	1.92(1.33–2.79)
+walk time, HOMA-IR, WHR, BMI,diabetes	1.00	1.26(0.81–1.95)	1.67(1.13–2.46)	1.49(1.01–2.19)

*The basic model included the following covariates: age, sex, education, current or past smoking and alcohol consumption, snoring, SBP, DBP, and duration of night sleep.

### Day napping duration, NAFLD, and inflammation

**Figure 2:**
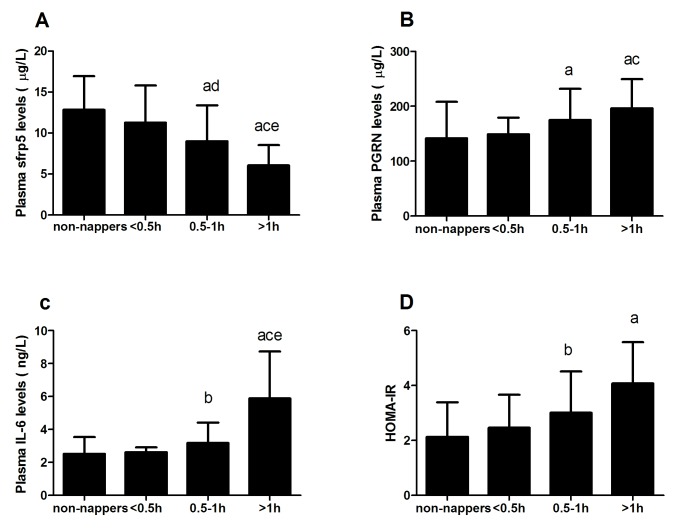
Circulating SFRP5, PGRN, IL-6, and HOMA-IR levels in cohort 2 population according to duration of day napping. Data are presented as means±SD. The *P* trend <0.001 for SFRP5, PGRN, IL-6, and HOMA-IR according to the duration of day napping. To analyze the statistical significance for a linear trend, these four variables were divided into tertiles. ^a^ *P*<0.01 compared with non-nappers, ^b^ *P*<0.05 compared with non-nappers, ^c^ *P*<0.01 compared with day napping duration <0.5 h, ^d^ *P*<0.05 compared with day napping duration <0.5 h, ^e^ *P*<0.01 compared with day napping duration 0.5–1 h.

In cohort 2, to determine whether inflammatory factors affected the relationship between day napping and NAFLD, we first verified the levels of pro- and anti-inflammatory factors in different groups by the duration of day napping. The longer day nappers (0.5–1 h and >1 h) had higher levels of PGRN, IL-6, HOMA-IR and lower SFRP5 levels (Figure 2, all *P*<0.05 and *P* for trend <0.01) compared with non-nappers. However, there were no significant differences in PGRN, IL-6, HOMA-IR and SFRP5 between the short nappers (<0.5 h) and non-nappers (Figure 2, all *P*>0.05). Then, we set NAFLD as the dependent variable in the multivariate logistic regression analyses, including the basic model covariates (described above), walking time, WHR, BMI, and diabetes. We found that a longer day napping duration (0.5–1 h, OR, 4.33 [95% CI, 1.48–12.75] and >1 h, OR, 7.42 [95% CI, 2.24–24.61]) was related to a higher prevalence of NAFLD compared with non-nappers. However, the association between day napping duration and NAFLD disappeared when further adjusted for SFRP5, PGRN, IL-6, and HOMA-IR (all *P*>0.05, Table 4).

**Table 4:** Association of NAFLD with duration of day napping adjusted by inflammtory covariates in cohort 2.

	Duration of napping (h)			
	Non-napping	<0.5	0.5–1	>1
Basic model*	1.00	2.32(0.55–9.85)	4.39(1.51–12.76)	8.06(2.63–24.72)
+added model†	1.00	2.23(0.51–9.71)	4.33(1.48–12.75)	7.42(2.24–24.61)
+added model and HOMA-IR	1.00	0.98(0.13–7.41)	0.68(0.12–3.84)	0.52(0.09–2.81)
+added model and SFRP5	1.00	0.85(0.13–5.58)	0.53(0.12–2.31)	0.35(0.06–2.03)
+added model and PGRN	1.00	0.98(0.77–1.81)	4.29(0.27–68.31)	10.43(0.63–71.94)
+added model and IL-6	1.00	2.39(0.46–12.49)	1.60(0.41–6.19)	1.49(0.23–9.59)

*The basic model included the following covariates: age, sex, education, current or past smoking and alcohol consumption, snoring, SBP, DBP, and duration of night sleep.

† The added model including: walk time, WHR, BMI, diabetes. SFRP5, Secreted Frizzled-related Protein; PGRN, progranulin; IL-6, interleukin-6.

## Discussion

In cohort 1, a large-sample, cross-sectional study, we found that the duration of day napping was significantly associated with a higher prevalence of NAFLD among an elderly Chinese population, whereas these results were not found among nappers who took shorter naps, i.e., less than 0.5 h. After adjusting for sex, age, education, snoring, past or current smoking, past or current drinking, SBP, DBP, night sleep duration, walking time, HOMA-IR, WHR, BMI, and diabetes, longer day napping duration (0.5–1 h and >1 h) was still associated with NAFLD. In cohort 2, we found that inflammatory factors significantly affected the association between day napping duration and NAFLD.

The prevalence of day napping in our study is 23.1%, while Fang et al. found a 3 times higher prevalence of day napping in Dongfeng–Tongji cohort of retired workers. This may be because our participants came from community and possess different working background which leading to different habit of napping. Day napping is considered to be a healthy habit for younger people. It promotes wakefulness, enhances performance and learning ability, and it improves emotional states [20], [21]. However, in older people, habitual napping may be a risk factor for morbidity and mortality. Bursztyn et al. [22] found that the mortality in 70-year-old Jerusalem residents who were in the habit of taking a daytime nap was twice as high as that in residents who were non-nappers, independent of other factors. Many previous cross-sectional studies found an association between day napping and diabetes in elderly populations (>50 years). These studies drew the similar conclusion that habitual day napping is related to a higher risk of diabetes. Recently, Xu et al. [9] observed the same results in a large prospective study (OR, 1.55, 95% CI, 1.45–1.66). It is widely believed that diabetes and NAFLD share many common mechanisms in their pathogenesis and progression [13]. However, the association between day napping and NAFLD remains unknown. In cohort 1 of our study, we also found a higher prevalence of diabetes in the nappers, which was consistent with the findings from previous studies. We also found that habitual day nappers had a higher prevalence of NAFLD and higher levels of FLI.

The duration of napping is very important in the role of day naps as they relate to NAFLD. A number of investigators observed that short day naps of less than 0.5 h duration had an invigorating effect because short naps prevent people from reaching deep sleep. During a short nap, people can go from stage I sleep to rapid eye movement (REM) sleep and wake up refreshed without having gone into a deeper sleep, from which it would be difficult to awaken [23]. Moreover, short nappers are less likely to experience sleep inertia, the impaired alertness and performance that last for approximately 30 minutes after awakening from a nap [24], [25]. Recently, Fang et al. [6] reported that only napping longer than 30 minutes was associated with a higher risk of diabetes. In our study, we found a significantly higher prevalence of NAFLD in only the long-duration (>0.5 h) habitual day nappers compared with non-nappers. The prevalence of NAFLD was higher in the shorter nappers (<30 min), but the result was not statistical significant. In the longer nappers, after adjusting for diabetes and other potential factors, the association with day napping duration was attenuated but still significant. It is indicated that longer, habitual day napping duration may be a risk factor for NAFLD independent of diabetes and other factors such as lipid levels, insulin resistance, BMI, and WHR.

The mechanism for the association between longer day napping and NAFLD is unclear. Chronic inflammation is considered to be one of the most important factors in NAFLD pathogenesis according to the “two-hit” hypothesis [26]. In the first hit (triglyceride accumulation in hepatocytes), increased levels of pro-inflammation factors and decreased levels of the anti-inflammation factors secreted by the adipose tissue are associated with hepatic lipid accumulation, metabolic alterations, and the development of hepatic steatosis. In the second hit, the imbalance of inflammatory cytokines leads to fibrosis and collagen deposits, the dysregulation of lysosomal metabolism, and endoplasmic reticulum stress, leading to apoptotic and necrotic cell death [27]–[29]. Moreover, day napping is also believed to involve inflammatory factors. In earlier studies, the daytime levels of IL-6 increased after total or partial sleep loss and tended to decrease during the compensatory day napping [30], [31]. Moreover, the pro-inflammatory cytokines IL-6 and TNF-α have been suggested to be mediators of excessive sleepiness in humans with pathologic conditions, e.g., sleep apnea [32] and narcolepsy [33], and in experimentally induced sleepiness [30], i.e., following sleep deprivation. Alves et al. [34] found that people with sleep apnea which manifested as excessive daytime sleepiness had elevated levels of inflammatory cytokines (CRP, IL-6, and TNFα), and those pro-inflammatory factors were improved by physical exercise. Although these previous studies did not conclude that there was a causal relationship between habitual day napping and inflammatory factors, we can at least conclude that day napping had a strong association with inflammatory factors. In cohort 1, day nappers had higher prevalence of snoring and we speculated this higher prevalence may explain part of the higher levels of pro-inflammatory factors. However, after adjusted by snoring, longer day napping duration were still associated with NAFLD. It is indicate that there are other factors involve in the association. In cohort 2 of our study, we first analyzed the levels of pro-inflammatory cytokines (IL-6, PGRN) and anti-inflammatory cytokine (SFRP5) in a small sample population, and we found that the longer habitual day nappers had higher levels of IL-6 and PGRN and lower levels of SFRP5 compared with non-nappers. However, there were no difference between shorter day nappers (<0.5 h) and non-nappers. We also found that the association between longer habitual day napping and the prevalence of NAFLD was no longer statistically significant after adjusting for IL-6, PGRN, and SFRP5. We could speculate that these inflammatory cytokines were an essential link between habitual day napping and NAFLD, but more researches are needed to confirm this link and elucidate the mechanisms.

There are some limitations in our study. First, this study is limited by its cross-sectional design and does not imply a causal connection among day napping, inflammatory factors, and NAFLD. Second, in our study, we diagnosed NAFLD primarily depending on FLI, but the gold standard method, liver biopsy, cannot be used in epidemiological studies in mostly healthy subjects for ethical reasons. Non-invasive examinations such as ultrasound are considered good methods for diagnosing of NAFLD, but they have some issues. Detecting hepatic fat via ultrasound has a threshold of above 30%, but for MRI, the limit is 5% [35]. Moreover, these methods were restricted by our large sample size. Third, we did not collect information on the timing of day napping. Some day nappers might take more than one nap, and some participants who reported no napping occasionally did take naps. These situations might have caused an overestimation or underestimation, but not a reversal, of the association of day napping with the prevalence of NAFLD.

To our knowledge, this was the first report to describe the association between day napping duration and NAFLD. Our study revealed that longer habitual day napping was associated with a higher prevalence of NAFLD in an elderly Chinese population and that the association may be affected by inflammatory cytokines. These results may provide important references for the elderly when they decide how long to nap during the day. However, perspective or mechanistic studies are needed to confirm these findings and elucidate the underlying mechanisms in the future.
